# Bed bug aggregation on dirty laundry: a mechanism for passive dispersal

**DOI:** 10.1038/s41598-017-11850-5

**Published:** 2017-09-28

**Authors:** William T. Hentley, Ben Webster, Sophie E. F. Evison, Michael T. Siva-Jothy

**Affiliations:** The Department of Animal and Plant Sciences, The University of Sheffield, Sheffield, UK

## Abstract

Bed bugs have shown a recent and rapid global expansion that has been suggested to be caused by cheap air travel. How a small, flightless and anachoretic insect that hides within its host’s sleeping area manages to travel long distances is not yet clear. Bed bugs are attracted to the odour of sleeping humans and we suggest that soiled clothing may present a similarly attractive cue, allowing bed bugs to ‘hitch-hike’ around the world after aggregating in the laundry bags of travellers. We show that (1) soiled clothing is significantly more attractive than clean clothing to active bed bugs moving within a bedroom sized arena and (2) elevation of CO_2_ to a level that simulates human occupancy in the same arena appears to initiate search behaviour rather than direct it. Our results show, for the first time, how leaving worn clothing exposed in sleeping areas when travelling can be exploited by bed bugs to facilitate passive dispersal.

## Introduction

The common bed bug *Cimex lectularius* L. has recently undergone a global resurgence^1^ which has been partly attributed to the increase in low cost international travel^2^. How a small, flightless, anachoretic (living in crevices or holes^3^) insect that prefers to hide in the sleeping area of its host disperses over long distances is unclear. We know bed bugs are able to actively disperse on a local scale^4^ because, for example, infestations in the same multi-occupancy building usually consist of closely related populations established from a single founding event^5, 6^. One possible mechanism facilitating long-range dispersal is that the insects find their way into clothing and/or luggage – the ‘vehicle’ – that then allows them to accompany the host to a new refuge^2^. The ability of, and preference for, bed bugs to use established hiding places is well documented^1, 7^ and is an important phenomenon in the problems associated with their control. However, it appears to be at odds with an ability to sequester and hide themselves in ‘novel’ hiding places such as suitcases and clothes in order to facilitate passive dispersal.

In contrast to their dispersal mechanisms, the host-seeking behaviour of the bed bug (and other haematophagous insects^8^) has received considerable empirical attention^9^ and is known to involve a combination of cues including host thermal, olfactory and visual signals^10–12^ with CO_2_ playing an important modulatory role^12^. In mosquitos, elevated CO_2_ stimulates foraging behaviour^13^ by activating host-seeking and directing flight towards the CO_2_ source^14^. However, when human odour is also present, the mosquito ignores the CO_2_ plume and navigates towards the odour source^15^, suggesting CO_2_ functions as a cue that activates host searching rather than directing it. Similar effects may operate in bed bugs^11^. Human odour is thought to attract bed bugs since it elicits both electrophysiological^16^ and behavioural responses^11^. Potential ‘vehicles’ for passive dispersal, such as luggage, are likely to contain recently worn clothes (i.e. those soiled with sweat and volatiles) that release human odour, especially since travellers tend to take home their dirty laundry. Odours from soiled clothing (or luggage containing soiled clothing) may therefore influence host-searching behaviour in bed bugs and consequently facilitate the passive dispersal of bed bugs *via* long-distance transport networks. We used a bedroom-scale experimental arena to determine whether (a) bed bugs would leave their refugia, (b) bed bugs would aggregate on soiled clothing, and (c) whether elevated CO_2_ (to simulate a human host in the room) modulated bed bug behaviours.

## Results

**Figure 1:**
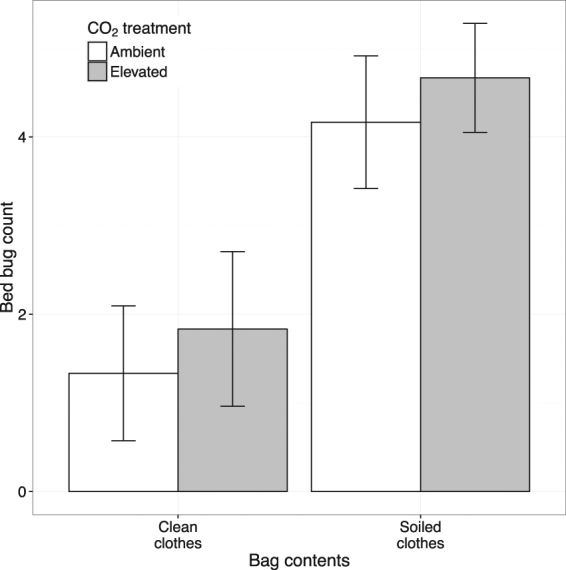
Number (mean ± S.E.) of bed bugs found in or on bags containing clothes with or without human odour, n = 6.

Bed bugs were most likely to be on/in bags containing soiled clothes than on/in bags containing clean clothes (Table 1a, Fig. 1). Elevated CO_2_ had no effect on this result (Fig. 1).

**Table 1:** Generalised Mixed Effect Model results summary for (**a**) bed bug distribution in relation to their location in the arenas and the presence of a CO_2_ source. Of the bed bugs found on clothes, (**b**) bed bug distribution in relation to their gender, human odour treatment and the presence of a CO_2_ source. Interaction and main effects were evaluated using log-likelihood ratio tests. Entries in bold are statistically significant, only significant interactions shown, n = 6.

	FIXED EFFECT	χ^2^	D.F.	P
a) *CLOTHING CHOICE*	Bed bug sex	1.44	1	0.230
	**Odour treatment**	**24**.**11**	**1**	**<0**.**0001**
	CO_2_ treatment	0.445	1	0.504
b) *ROOM DISTRIBUTION*	**Location in room**	**23**.**87**	**2**	**<0**.**0001**
	CO_2_ treatment (CO_2_)	1.35	1	0.245
	Location in room * CO_**2**_	**11**.**77**	**2**	**0**.**003**

**Figure 2:**
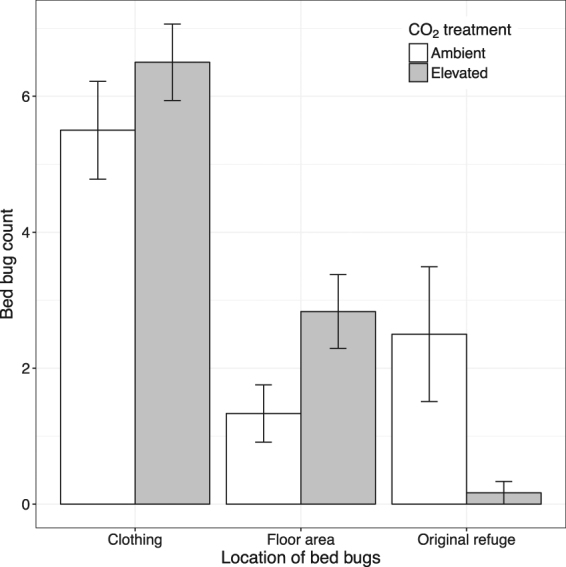
Distribution (mean ± S.E.) of bed bugs within the experimental arena without (white) and with (grey bars) a CO_2_ source, n = 6. Original refuge refers to corrugated filter paper on which the bed bugs were introduced into the arena. Floor is any location within the arena that is not clothing or original refuge.

However, there was a significant interaction between CO_2_ treatment and the distribution of bed bugs within an arena (Fig. 2). In the presence of elevated CO_2_ more bed bugs left the refuge (Table 1b, Fig. 2) with only 1.6 ± 1.6% of the bed bugs remaining in the refuge. By contrast, significantly more (25 ± 9.9%) bugs were in the refuge at the end of the ambient CO_2_ trial (Table 1b, Fig. 2). The extra dispersing individuals under elevated CO_2_ were not found aggregating on clothing (Fig. 2), but instead, were found on the floor within the arena such as adjacent to the arena perimeter or, on three occasions, the open floor area. There was no difference in the number of males and females that dispersed within the room (Table 1a). The position of bed bugs in the room was not influenced by the location of the CO_2_ source (χ^2^_(11)_ = 10.01, *p* = 0.53).

## Discussion

In the absence of a human host, bed bugs were twice as likely to aggregate on bags containing soiled clothes compared to bags containing clean clothes. Contrary to our predictions, elevated CO_2_ did not affect this result. We did not observe bed bugs aggregating on the side of the room with the CO_2_ source, but instead found an increased likelihood of bed bugs leaving their refuge. Our data are most consistent with the notion that elevated CO_2_ initiates host-seeking behaviour. This is similar to findings in *Aedes aegypti* and *Anopheles gambiae* where very small increases in CO_2_ concentration stimulate host-seeking behaviour^19, 20^. The position of the CO_2_ source in a closed room above and away from the bed bug release point means directional cues as to the source of CO_2_ may have been difficult for bed bugs to discern and so it is not possible to conclude whether or not the lack of aggregation on that side of the room suggests a lack of attraction to CO_2_. Many commercial bed bug monitors use CO_2_ in conjunction with thermal and/or chemical cues, and claim higher catch rates than monitors without CO_2_. Our results suggest that any increase in capture rate in such devices^10, 21^ might be caused by increased activity rather than, or in addition to, increased attractiveness.

The smallest bottle-neck through which a new bed bug population can pass, and therefore the most likely passive (i.e. long-range) dispersal phenotype, is a single mated adult female^5^. There is some experimental evidence for a female ‘disperser’: work using small arenas and shorter time scales found that females were more active than males^22^ and more likely to disperse from aggregations with a 1:1 sex ratio^23^. By contrast, our study showed there was no significant sex difference in the number of insects found on bags with, or without, human smell (Table 1a). Since dispersal from the refuge may be initiated by hunger, we would expect females (with their higher metabolic rate^24^) to start host-seeking before males and potentially to do so more often. However, over longer periods, such as that used in this study, males and females are both likely to experience hunger and disperse from the refuge. Our design does not enable us to resolve this issue.

Our results do show that bags containing soiled clothes were significantly more attractive to bed bugs than identical bags containing clean clothes. Human odour is a known cue for host-seeking bed bugs^11^, but until now studies have focussed on how these cues determine the attractiveness of a sleeping host. Our results show that bed bugs will move to, and aggregate on, soiled clothing in the absence of a host. Bed bugs can sense 104 different volatiles^16^ found in the odorant profile of human skin^25^. Many of these volatiles are likely to evaporate from ‘soiled’ clothes for some time after they have been worn, removed and/or stored. We propose that residual human odour on soiled clothes acts as an elicitor of host-seeking or host-seeking-like behaviours, and that cues such as heat^9^ or elevated CO_2_ may facilitate the activation and sensitivity of such host-seeking behaviour. Consequently, soiled clothing left in an open suitcase, or left on the floor, of an infested room is likely to attract bed bugs. When packed into the suitcase, they will accompany their host back home.

Our results show that over a period of several days bed bugs are attracted to, and remain on, soiled clothing: this provides a biologically realistic mechanism that underpins passive, long-range dispersal in bed bugs. The advent of relatively frequent, short-stay holidays in locations long distances from the hosts’ residence will facilitate the proposed mechanism of dispersal. Careful management of holiday clothing may be an important strategy in the prevention of bringing home bed bugs.

## Method

### Experimental production of soiled clothing

Four volunteers were chosen at random from a pool of eight for each run of the experiment. They washed using a non-perfumed soap (Simple® soap, Unilever, UK) and then wore a white, 100% cotton, t-shirt and socks (Sportee range, Decathlon, UK), for three hours during normal daily activity between 12:00 and 18:00. The clothes were immediately sealed in an airtight bag (Ziploc™, 18 cm × 19 cm) and were used in the experiment within 24 hours of being worn. Clothes and cotton bags (see below) were washed (90 °C) with a non-perfumed detergent (Surcare™ Non-Biological laundry liquid – ingredients include 5–15% non-ionic surfactants, anionic surfactants, soap, < 5% phosphonate) between trials. Clothes were placed in clean cotton tote-bags (38 × 43 cm, Clever Baggers Ltd, UK) and were washed after every use.

### Experimental rooms

Experiments were conducted simultaneously in two identical, temperature-controlled rooms (4.8 m (L) × 4.3 m (W) × 2.4 m (H)). Each room was maintained at 22 °C ± 0.5 °C, 55% ± 10% relative humidity and 12 h L:D with a 30-minute ramping period to simulate dawn and dusk. Ventilation within each room provided six complete air changes per hour. During each experiment one room was designated the ‘elevated CO_2_ treatment’ room and received an increase in CO_2_ concentration in the centre of the arena at ground level by 28 ± 1 ppm (Fig. S1) to simulate a situation where a human is breathing away from the clothing, (a single inactive adult sitting in the same room −1.5 m from the centre - increased the CO_2_ concentration by *ca*. 14 ± 1 ppm at the centre). CO_2_ was generated by placing approx. 2 kg dry ice in a polystyrene container (25 × 25 × 23 cm) 1.5 m from the centre of the room. Dry ice was replaced daily during the light-phase. The other room was designated the ‘ambient CO_2_ treatment’ and had a polystyrene container with no dry ice.

Four clean cotton tote bags, each containing one t-shirt and one pair of socks (two bags with soiled clothes, two with clean clothes), were placed 1 m from the room centre at 90 degrees from each other (Fig. S1) alternating between clean and soiled contents. Therefore, bags containing the same treatment were always opposite each other. The cross-shape formation of the bags (Fig. S1) was maintained at all times, but rotated around a virtual clock face. The position of the first bag was chosen at random from numbers 1–12 using a random number generator (Fig. S1), which then in turn determined the position of the other bags. Between each experimental run the floor was cleaned using bleach (Mexcel®, SLS, UK) and remained empty for 24 hours before the next experimental run.

### Experimental procedure

Bed bugs were fed to satiation on a human blood meal 24 hours prior to being placed in the arena. A standard laboratory rearing container for bed bugs (a 60 ml clear acrylic pot with 5 cm × 20 cm corrugated filter paper – henceforth ‘the refuge’) containing 5 male and 5 female adult bed bugs was placed under a 10 × 10 × 10 cm clear plastic box in the centre of the room for 48 hours (to prevent the bugs dispersing as a potential escape response). Four clean tote-bags (containing the clean and soiled clothes) were then introduced into the room. Twenty-four hours later the clear plastic box over the refuge was removed (giving the bugs unhindered physical and sensory access to the room). No interim measurements were taken and the arena was observed via a viewing window. After a further 96 hours, the number and location of each bed bug was recorded. Location was categorised as remaining in the original refuge, within/on clothing bag, or on the floor of the arena. The experiment was repeated six runs, alternating which room was designated to ambient or elevated CO_2_.

### Statistical analysis

Analysis was performed with R version 3.3.1 (R Core Team 2016). Using a GLMM^17^ with a binomial error distribution, the response variable bed bug count, was generated as a proportion of the number of available bed bugs – number found at different locations within the room. This method accounted for any individual that died (5 over the six runs). To account for uncontrolled variation within the rooms (e.g. slight variation in lighting or ventilation), room and experimental run were used as nested random terms within the model. Minimum adequate models were determined from stepwise exclusion of parameters from a full model using log-likelihood tests^18^.

### Data availability

The data that supports the findings of this study are available via The University of Sheffield Online Research Data Archive.

## Electronic supplementary material

Supplementary figure
