# Supplementary material for: Bed bug aggregation on dirty laundry: a mechanism for passive dispersal
Source: Sci Rep. 2017 Sep 28;7:11668. doi: 10.1038/s41598-017-11850-5 (PMC5620066; doi:10.1038/s41598-017-11850-5)

Bed bug aggregation on dirty laundry: a mechanism for passive dispersal

William T. Hentley*, Ben Webster, Sophie E. F. Evison, Michael T. Siva-Jothy

The Department of Animal and Plant Sciences, The University of Sheffield, Sheffield, UK.

*Corresponding author

Figure S1. Schematic of experimental arena. Tote bags containing the same treatment were always opposite each other. The cross-shape formation of the bags was maintained at all times, but rotated around a virtual clock face (numbers around the outside of the arena). The position of the first bag was chosen at random from numbers 1-12 using a random number generator, which then in turn determined the position of the other bags. In this example, the first bag, “Bag 1”, was placed in line with 6 o’clock which determined the positon of the other bags. Other features in the room include: (a) Cotton tote-bag containing clothes, (b) bed bug original refuge / release point, (c) vertical barrier preventing escape and (d) dry-ice container. Not to scale.

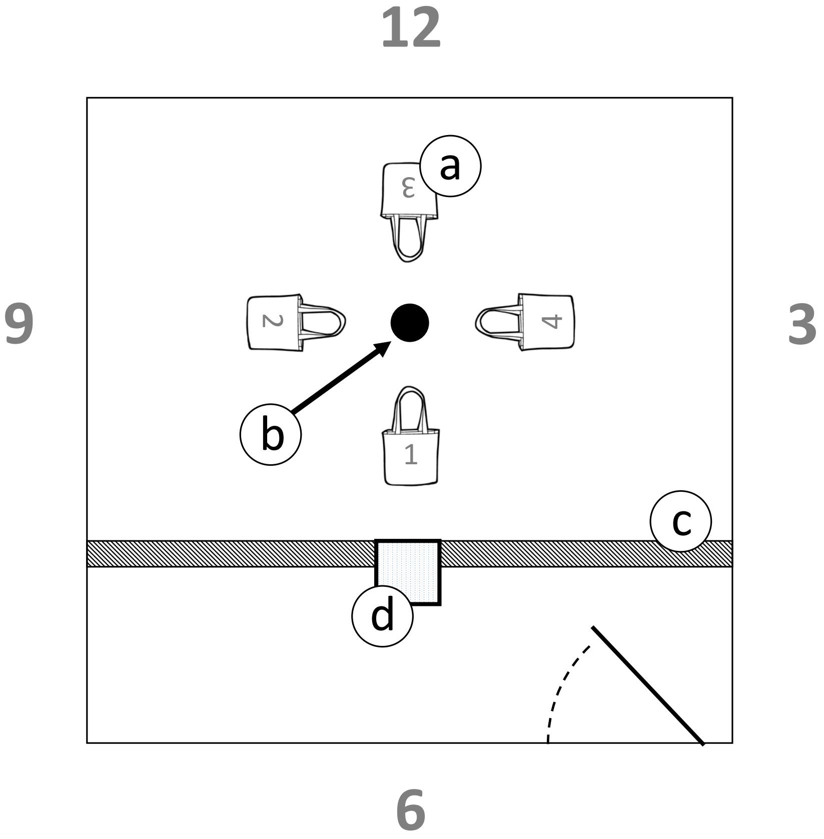

Supplement: Supplementary file 1 — Supplementary figure [file 41598_2017_11850_MOESM1_ESM.pdf]
